# Adding Stiffness to the Foot Modulates Soleus Force-Velocity Behaviour during Human Walking

**DOI:** 10.1038/srep29870

**Published:** 2016-07-15

**Authors:** Kota Z. Takahashi, Michael T. Gross, Herman van Werkhoven, Stephen J. Piazza, Gregory S. Sawicki

**Affiliations:** 1 Department of Biomechanics, University of Nebraska at Omaha, USA; 2 Division of Physical Therapy, University of North Carolina at Chapel Hill, USA; 3 Department of Health and Exercise Science, Appalachian State University, USA; 4 Department of Kinesiology, The Pennsylvania State University, USA; 5 Joint Department of Biomedical Engineering, University of North Carolina at Chapel Hill and North Carolina State University, USA.

## Abstract

Previous studies of human locomotion indicate that foot and ankle structures can interact in complex ways. The structure of the foot defines the input and output lever arms that influences the force-generating capacity of the ankle plantar flexors during push-off. At the same time, deformation of the foot may dissipate some of the mechanical energy generated by the plantar flexors during push-off. We investigated this foot-ankle interplay during walking by adding stiffness to the foot through shoes and insoles, and characterized the resulting changes in *in vivo* soleus muscle-tendon mechanics using ultrasonography. Added stiffness decreased energy dissipation at the foot (p < 0.001) and increased the gear ratio (i.e., ratio of ground reaction force and plantar flexor muscle lever arms) (p < 0.001). Added foot stiffness also altered soleus muscle behaviour, leading to greater peak force (p < 0.001) and reduced fascicle shortening speed (p < 0.001). Despite this shift in force-velocity behaviour, the whole-body metabolic cost during walking increased with added foot stiffness (p < 0.001). This increased metabolic cost is likely due to the added force demand on the plantar flexors, as walking on a more rigid foot/shoe surface compromises the plantar flexors’ mechanical advantage.

Recent evidence suggests that the foot and ankle embody fundamental structure-function relationships at play during human locomotion. For example, during push-off from the ground, the foot functions as a lever with lever arms for input forces (ankle plantar flexors) and output forces (those originating from the ground). At each step during walking and running, forces underneath the foot propagate from heel to toe^1^,^2^,^3^, creating a continuously changing ratio between these lever arms, or “gear ratio”^4^. This gearing mechanism afforded by the foot influences not only muscle-tendon leverage, but also muscle-tendon force-generating capacity through modulation of fibre shortening velocity^4^,^5^. The ankle plantar flexors work within these geometric constraints to achieve favourable operating points on the force-length^6^ and force-velocity profiles^7^,^8^,^9^, and generate a burst of positive power during push-off. In fact, the muscle-tendon units of the ankle perform more positive work during walking and running than those of any other joint including the knee and hip^10^,^11^, contributing to forward propulsion^12^ and increasing whole-body mechanical energy^13^.

While foot gearing appears to aid in generation of plantar flexor force and ankle push-off power, recent studies have shown that the foot is a potential energy sink during locomotion. In fact, much of the energy generated by the ankle muscle-tendon structures during push-off is absorbed by and dissipated through foot deformation^14^,^15^, including extension of the metatarsal-phalangeal joints in late stance^16^,^17^,^18^. When humans walk faster, the ankle muscle-tendon units generate more energy while the foot absorbs more energy^19^. Further investigation of this opposing energetic interplay may elucidate the underlying mechanisms that regulate mechanics and energetics of human locomotion. Such insights may lead to improved understanding of foot and ankle pathologies, and inspire novel designs for wearable devices, such as prostheses, orthoses, exoskeletons, and footwear.

A potentially valuable approach to studying interactions between the foot and ankle is to alter the structural properties of the foot/shoe interface, and analyse the resulting changes in ankle function. For example, prescribing shoes with increased midsole longitudinal bending stiffness can alter foot mechanics in several ways. First, the added bending stiffness would act to restrict extension at the metatarsal-phalangeal joints, and this effect is expected to reduce the magnitude of energy dissipation in this foot region^20^,^21^. Second, restricting metatarsal-phalangeal joint extension would enable the ground reaction force to travel farther away from the ankle joint centre, which would lengthen the output lever arm and increase the gear ratio of the foot and ankle complex^22^. Previous studies of human running support these expectations, where increasing foot/shoe stiffness has been found to reduce foot energy dissipation^21^ and increase the gear ratio^22^. While increased gear ratio might be expected to enhance plantar flexor force by slowing the speed of muscle-tendon contraction^4^,^5^,^23^, no *in vivo* studies of muscle-tendon mechanics during walking or running have confirmed this.

The purpose of this study was to examine the interplay between the foot and the muscle-tendon dynamics of the ankle plantar flexors during walking by systematically increasing the bending stiffness of the foot. We used insoles of varying thickness and shoes to increase midsole longitudinal bending stiffness. The shoes/insoles, however, were not necessarily intended to modify the stiffness of the anatomical foot itself. By increasing bending stiffness, we aimed to make the foot/shoe complex more rigid to inhibit energy dissipation and to lengthen the foot’s output lever arm, and thus increase the gear ratio. In each walking trial, we used ultrasound imaging to study the soleus muscle fascicle contractile behaviour, and we used indirect calorimetry to analyse whole-body metabolic energy expenditure. We hypothesized that the larger gear ratio that follows from added foot stiffness would increase soleus force and decrease soleus fascicle shortening velocity. Additionally, we hypothesized that decreased soleus shortening velocity would enable more economical force production and thus reduce whole-body metabolic energy expenditure during walking.

## Results

### Added Foot Stiffness

From a three-point bending test, it was determined that shoes and three different thickness of carbon fibre insoles (0.8, 1.6, and 3.2 mm) resulted in midsole longitudinal bending stiffness values of: 14.8 ± 0.5, 22.5 ± 0.5, 28.7 ± 0.8, and 65.6 ± 2.9 N/mm, respectively (mean ± standard error). These values represented the magnitude of added stiffness (ΔK) relative to the barefoot condition, and signify the resistance to bending motion in the forefoot. Our stiffness values are within the range of values reported in a previous study^20^, where investigators used control shoes and two different layers of carbon fibre insoles that yielded stiffness values of: 18, 38 and 45 N/mm, respectively^20^.

### Foot Deformation Power and Gear Ratio

**Figure 1:**
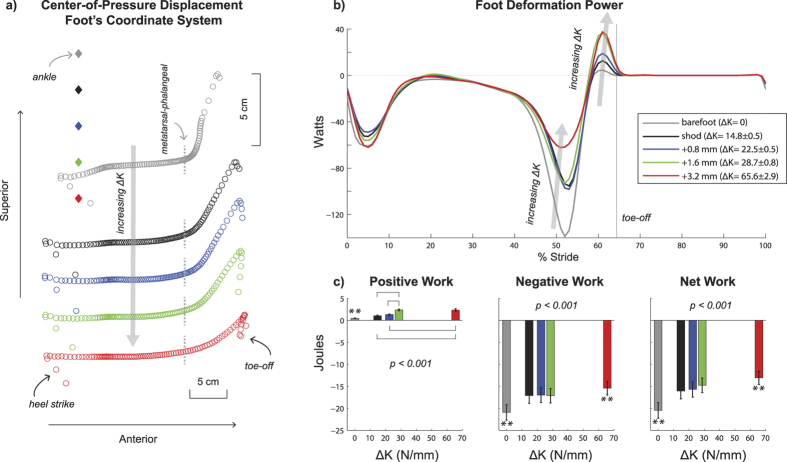
Adding stiffness to the foot altered the centre-of-pressure propagation during stance, and decreased net mechanical work due to foot deformation. (**a**) The centre-of-pressure (COP) during stance was expressed in the foot’s sagittal plane (N = 20). Each circle corresponds to averaged COP data for one percent of stance. The position of the ankle joint in the foot’s reference is denoted by the diamonds. A vertical projection of the metatarsal-phalangeal joint in the foot’s reference frame is denoted by the dashed vertical grey lines. (**b**) The foot deformation power was time-normalized to the stride cycle (N = 20). Three distinct phases of power were evident: negative power immediately after heel strike, negative power during ~30–60 percent of stride, and positive power before toe-off. (**c**) Total positive work, negative work, and net work were quantified during stride (N = 20, means ± s.e.m). With added foot stiffness (ΔK), there was an increase in total positive work (p < 0.001), decrease in magnitude of negative work (p < 0.001), and decrease in magnitude of net work (p < 0.001). P-values indicate the main effect of added foot stiffness. **denotes significant pair-wise difference with respect to each of the other conditions. Square brackets show additional significant pair-wise comparisons.

Added foot stiffness (ΔK) reduced the magnitude of energy dissipation during walking (Fig. 1). Foot deformation power was mostly negative for the majority of stance phase across all conditions, with a relatively small burst of positive power prior to toe-off. With added foot stiffness, there was a significant decrease in magnitude of negative work (p < 0.001) and a significant increase in positive work (p < 0.001). Most notably at the greatest foot stiffness condition (ΔK = 65.6 N/mm), magnitude of negative work was less than all other conditions, including a 26.3% and a 9.8% reduction compared to the barefoot and the least stiff shod (ΔK = 14.8 N/mm) conditions, respectively. The positive work for the greatest foot stiffness condition (ΔK = 65.6 N/mm) was greater than all other conditions, including a 398.7% and a 126.5% increase compared to the barefoot and the least stiff shod (ΔK = 14.8 N/mm) conditions, respectively. The changes in positive and negative work contributed to a significant decrease in the magnitude of net work (p < 0.001) with added foot stiffness (i.e., net work was less negative). In the greatest foot stiffness condition (ΔK = 65.6 N/mm), the magnitude of net work was less compared to all other conditions, including a 36.1% and a 18.6% lower magnitude relative to the barefoot and the least stiff shod (ΔK = 14.8 N/mm) conditions, respectively.

**Figure 2:**
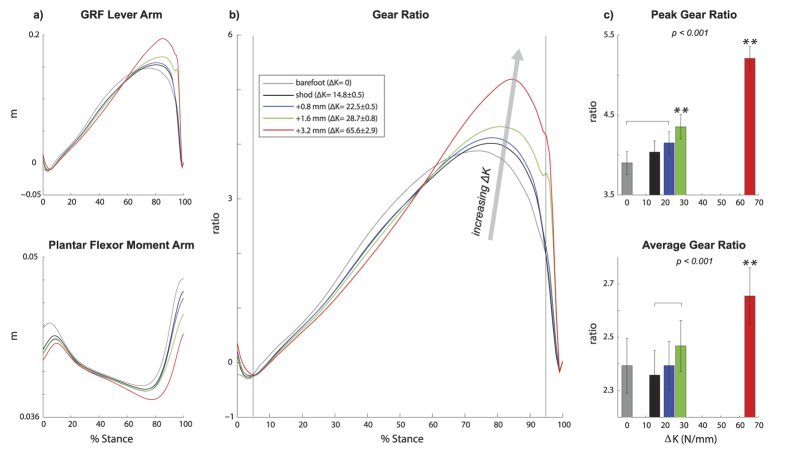
Adding stiffness to the foot increased the gear ratio during stance. (**a**) Lever arm of the ground reaction force (GRF) relative to the ankle, and moment arm of the plantar flexor during stance were time-normalized to percentage of stance (N = 20). (**b**) The gear ratio was estimated as the ratio of GRF lever arm to plantar flexor moment arm during stance (N = 20). The vertical lines define 5 and 95% of stance. (**c**) The peak gear ratio and the average gear ratio were computed during 5–95% of stance (N = 20, mean ± s.e.m). With added foot stiffness (ΔK), there were increases in the peak gear ratio (p < 0.001) and the average gear ratio during stance (p < 0.001). P-values indicate the main effect of added foot stiffness. **denotes significant pair-wise difference with respect to each of the other conditions. Square brackets show additional significant pair-wise comparisons.

The centre-of-pressure travelled farther forward along the foot (i.e., anterior) with added foot stiffness (Fig. 1), resulting in a greater gear ratio (Fig. 2). Both the ground reaction force lever arm and the plantar flexor moment arm changed continuously throughout stance for all conditions, contributing to a non-constant gear ratio (i.e., ratio of ground reaction force lever arm and plantar flexor moment arm) (Fig. 2). With added foot stiffness, the ground reaction force lever arm increased due to a more anterior propagation of the centre-of-pressure, while the plantar flexor moment arm decreased due to greater ankle dorsi flexion (Supplementary Fig. S1). These changes contributed to significant increases in peak gear ratio (p < 0.001) and average gear ratio during stance (p < 0.001). In the greatest foot stiffness condition (ΔK = 65.6 N/mm), the peak gear ratio was greater than all other conditions, including a 33.5% and a 29.0% increase compared to barefoot and the least stiff shod (ΔK = 14.8 N/mm) conditions, respectively.

### Soleus Muscle Activation, Force, Length, and Velocity

**Figure 3:**
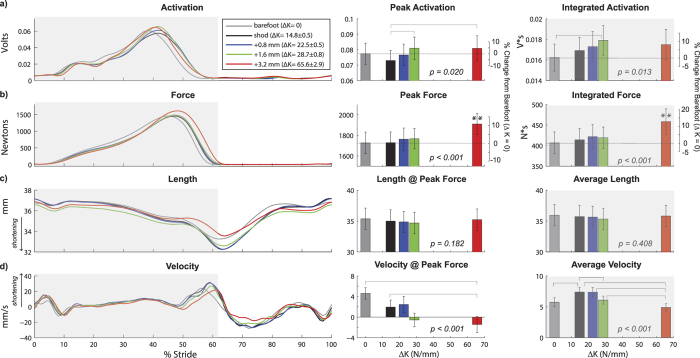
Adding stiffness to the foot increased soleus activation and force, and decreased fascicle shortening velocity. Time-normalized data (stride cycle) of (**a**) soleus activation (N = 19, left limb), (**b**) soleus force (N = 20, right limb), (**c**) fascicle length (N = 20, right limb), and (**d**) fascicle velocity (N = 20, right limb). Stance phase is highlighted in grey. With added foot stiffness (ΔK), there was an increase in peak soleus activation (p = 0.020), increase in integrated soleus activation during stance (p = 0.013), increase in soleus peak force (p < 0.001), and increase in soleus integrated force during stance (p < 0.001). In addition, added foot stiffness decreased soleus fascicle velocity at peak force (p < 0.001) and decreased the average fascicle shortening velocity during stance (p < 0.001) but had no significant effect on fascicle length at peak force (p = 0.182) and the average fascicle length during stance (p = 0.408). P-values indicate the main effect of added foot stiffness. **denotes significant pair-wise difference with respect to each of the other conditions. Square brackets show additional significant pair-wise comparisons.

Added foot stiffness significantly increased soleus muscle activation and force output (Fig. 3). Specifically, added foot stiffness increased peak activation (p = 0.020), in which the two greatest foot stiffness conditions (ΔK = 65.6 N/mm and ΔK = 28.7 N/mm) had 10.7% and 10.9% greater peak activation than the least stiff shod condition (ΔK = 14.8 N/mm), respectively. Integrated activation during stance also increased with added stiffness (p = 0.013), in which an intermediate stiffness condition (ΔK = 28.7 N/mm) was 10.3% greater than the barefoot condition. Peak force in the greatest added foot stiffness condition (ΔK = 65.6 N/mm) was greater than all other conditions, including a 10.7% increase relative to barefoot and a 10.5% increase relative to the least stiff shod condition (ΔK = 14.8 N/mm). Integrated force during stance was also greatest in the greatest added foot stiffness condition, with a 12.7% increase relative to barefoot and a 10.7% increase relative to the least stiff shod condition (ΔK = 14.8 N/mm).

**Figure 4:**
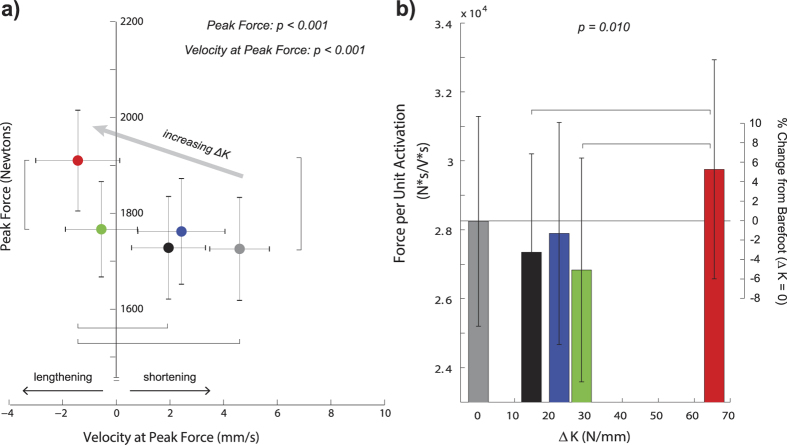
Slower fascicle shortening speed contributed to enhanced soleus force production. (**a**) Soleus peak force was plotted against fascicle velocity at the time of peak force (N = 20, mean ± s.e.m). With added foot stiffness (ΔK), there was a shift towards slower fascicle velocity at the time of peak plantar flexor force. At the greatest stiffness (ΔK = 65.6 N/mm), the soleus fascicle was actually lengthening during peak force production. P-values indicate the main effect of added foot stiffness, and significant pair-wise comparisons are denoted by the square brackets. (**b**) Force per unit activation was quantified as the ratio of integrated soleus force and integrated soleus activation during stance (N = 19, mean ± s.e.m). Added foot stiffness increased the force per unit activation (p = 0.010). The greatest added foot stiffness condition (ΔK = 65.6 N/mm) had greater force per unit activation compared to ΔK = 14.8 N/mm and ΔK = 28.7 N/mm (denoted by the square brackets).

With added foot stiffness, the force per unit activation (ratio of integrated force to integrated activation) was enhanced (p = 0.010) (Fig. 4). In the greatest foot stiffness condition (ΔK = 65.6 N/mm), force per unit activation was greater than it was for the two intermediate stiffness conditions, including an 8.8% increase relative to the ΔK  = 14.8 N/mm condition, and a 10.9% increase relative to the ΔK = 28.7 N/mm condition.

Added foot stiffness decreased soleus fascicle shortening velocity during stance (Fig. 3). For all foot conditions, the soleus fascicle shortened throughout most of stance, with shortening velocity reaching maximum approximately when the foot was leaving the ground. Added foot stiffness significantly reduced fascicle velocity at the time of peak force (p < 0.001), and average velocity during stance (p < 0.001). Velocity at peak force was close to zero for all conditions, and the greatest added foot stiffness condition (ΔK = 65.6 N/mm) had the slowest shortening velocity, including a difference of 6.0 mm/s relative to the barefoot condition and a difference of 3.4 mm/s relative to the least stiff shod condition (ΔK = 14.8 N/mm). Added foot stiffness had no significant effect on soleus fascicle operating length, including length at the time of peak force (p = 0.182) and average length during stance (p = 0.408).

### Whole-Body Net Metabolic Power

**Figure 5:**
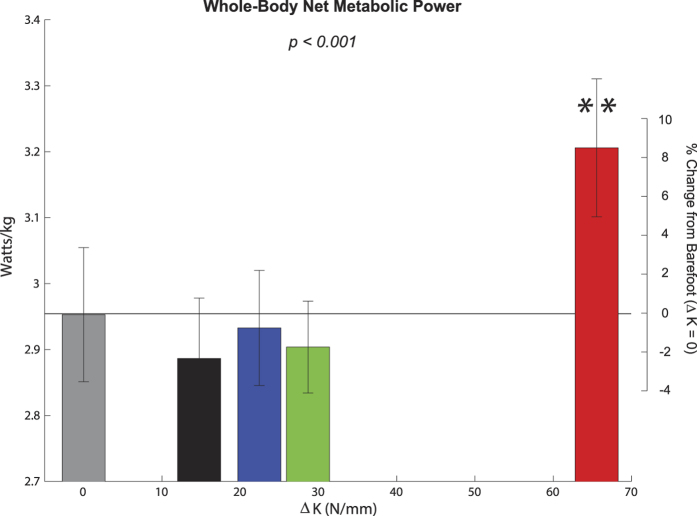
Whole-body net metabolic power was increased at the greatest added foot stiffness. Net metabolic power was quantified using indirect calorimetry (N = 20, mean ± s.e.m). P-value indicate the main effect of added foot stiffness. At the greatest added foot stiffness (ΔK = 65.6 N/mm), the net metabolic power was greater than each of the other conditions (denoted by**).

Walking with added foot stiffness increased net metabolic power (p < 0.001) (Fig. 5). For the greatest foot stiffness condition (ΔK = 65.6 N/mm), metabolic power was greater than all other conditions, including an 8.6% increase relative to the barefoot condition and a 11.1% increase relative to the least stiff shod condition (ΔK = 14.8 N/mm).

## Discussion

In this study, we added mechanical stiffness to the foot to alter its two salient functional features during walking: energy dissipation and gearing. Moreover, by making the foot/shoe complex more rigid, these structures dissipated less energy and increased the gear ratio. These results are consistent with similar studies addressing running with various shoe insoles^21^,^22^. The mechanism for these altered foot mechanics appears to be restricted mobility of the metatarsal-phalangeal joints^24^,^25^. These joints serve as the predominant source of energy dissipation during push-off^16^,^17^,^18^. Extension of these joints also serves to increase the ground reaction force lever arm, and thus the properties of foot energy dissipation and gearing may be inter-related. Stiffer feet/shoes are associated with less energy dissipation and with a greater gear ratio. With alterations in these foot mechanics due to added stiffness, we expected changes in the soleus muscle contractile behaviour during walking.

For all foot conditions, the soleus fascicles were largely shortening throughout the duration of stance, consistent with previous studies^9^,^26^. For most foot conditions, the soleus fascicles were either close to isometric or were shortening at the time of peak force, consistent with a previous study^9^. At greater added foot stiffness conditions, the contraction speed decreased such that the fascicles were actually lengthening at the time of peak force (Figs 3 and 4). This reduction in fascicle speed contributed to enhanced force production, indicating a shift in the force-velocity operating range. The mechanism for this force-velocity shift is likely due to an increase in gear ratio. Previous studies involving musculoskeletal modelling have shown that either an increase in ground reaction force (output) lever arm or a decrease in plantar flexor (input) moment arm, which can both increase the gear ratio, can contribute to a slower muscle-tendon shortening speed^23^,^27^. Interestingly, adding stiffness to the foot appears to affect both the output and input lever arms, through a more anterior propagation of the centre-of-pressure and altered ankle joint kinematics, respectively (Fig. 2, Supplementary Fig. S1). Thus, increase in ground reaction force lever arm and decrease in plantar flexion moment arm likely contributed to greater force production and slower fascicle speed with added foot stiffness.

To determine whether other factors besides speed of fascicle contraction could contribute to enhanced soleus force, we also examined soleus activation and fascicle operating length. The soleus muscle activation was greater with added foot stiffness, and thus we can attribute a portion of the force enhancement to greater muscle recruitment. However, force per unit activation was also greater with added foot stiffness. In other words, the relative change in force was greater than the change in muscle activation, such that the enhanced soleus force could not be explained by increased activation alone. Furthermore, adding stiffness to the foot had no significant effect on the soleus fascicle operating length, including the average length during stance and the instantaneous length at the time of peak force. Taking these results altogether, there is ample support for our hypothesis that adding foot stiffness enhances plantar flexor force production by reducing fascicle shortening speed.

Since slower fascicle contraction speed might be expected to permit more economical force production, we had hypothesized that the whole body metabolic cost would decrease with added foot stiffness. Contrary to our hypothesis, the metabolic cost was increased when walking with the greatest added foot stiffness (ΔK = 65.6 N/mm), including an 11.1% increase compared to the least stiff shod condition (ΔK  = 14.8 N/mm). This increase is comparable to walking with approximately 6 kg of added mass at the pelvis^28^. This elevated metabolic cost may be explained by the cost of producing greater force^29^,^30^. A rigid foot/shoe surface compromises the mechanical advantage of the plantar flexors, requiring increased force demand during push-off for these muscles. In other words, the metabolic demand to generate more force may have counterbalanced the potential energetic benefit of economical force production (i.e., shifting of force-velocity and enhanced force per unit activation). Interestingly, added foot stiffness also decreased the positive work generated by the plantar flexor muscle-tendon units (Supplementary Figs S1 and S2), despite greater force from the plantar flexors. These results may then support the idea that a key determinant of metabolic cost during locomotion is related to the muscle force production^29^,^30^.

Recent studies have also shown that changing the force demand on the plantar flexor could drive changes in metabolic cost. Off-loading the plantar flexors using an elastic ankle exoskeleton, for example, has been shown to reduce the metabolic cost of vertical hopping^31^ as well as walking^32^. Although compensations at other joints could certainly influence the whole-body metabolic cost, the changes in joint mechanics due to added foot stiffness appeared to be greatest at the ankle (Supplementary Figs S1 and S2). Thus, the resulting changes in the metabolic cost of walking is likely driven by the added force demand of the plantar flexors.

From this study alone, we cannot definitively rule out the possibility that the metabolic cost could be reduced at an intermediate stiffness. While there was no significant difference among barefoot and all other added foot stiffness conditions (other than the highest stiffness: ΔK = 65.7 N/mm), 10 of the 20 subjects had actually reduced the metabolic energy cost while walking under an intermediate stiffness condition (ΔK = 28.7 N/mm) compared to barefoot. This variable outcome could be, in part, due to morphological differences among the subjects. The structures of the human feet are highly variable, where person-to-person variations are prevalent in plantar flexor moment arm^33^, toe lengths^23^, arch height^34^,^35^, and plantar fascia thickness^36^. All of these factors could potentially influence the intrinsic stiffness of the bare foot, as well as magnitude of foot energy dissipation and gear ratio during walking. It is likely, then, that the way in which the muscles respond to added foot stiffness could vary across individuals. Future studies may need to account for the individual morphological variations, and determine if there is a subject-specific optimal level of added stiffness that could reduce metabolic cost.

With the capacity of foot structures to modulate plantar flexor function, harnessing this foot-ankle interplay could potentially enhance locomotion performance. While this study did not reveal metabolic benefit during walking with added foot stiffness, it is possible that the enhanced plantar flexor force could be beneficial in other locomotion tasks, especially when the unequivocal goal of the movement is to maximize the body’s acceleration. Sprinters for example, can have unique morphological characteristics such as a small plantar flexor moment arm and long toes that could enhance force production that may be advantageous during the start of a race^23^,^27^. Adding stiffness to the foot could achieve similar benefits to the sprinters’ foot, as increased gear ratio could achieve greater plantar flexor force. In fact, shoes with increased midsole longitudinal bending stiffness have been shown to augment sprinting speed^37^ and even enhance maximum jumping height^38^. Improving locomotion economy, on the other hand, appears more difficult. Roy and Stefanynshyn found that adding stiffness to shoes offers a very small metabolic benefit (~1%) during submaximal running^20^. Perhaps the difficulty in reducing metabolic cost during submaximal running or walking may be because human muscles are already performing with great efficiency^39^,^40^,^41^. The ankle plantar flexors, in particular, operate in favourable regions of force-length and force-velocity profiles during walking and running^6^,^7^,^8^,^9^. Adding stiffness to the foot during these submaximal tasks could then do more to elevate the plantar flexor force demand to overcome the compromised mechanical advantage, rather than to promote more economical force production (i.e., increased force per activation due to a favourable shift in force-velocity operating range).

To improve locomotion economy with added foot stiffness, a more plausible approach may be to target movement tasks where muscles operate with less economy, or tasks that require greater muscle force. At fast walking speeds close to the walk-to-run transition, for example, the force-generating capacity of the ankle plantar flexors diminishes due to faster shortening speeds^8^,^9^,^42^. Also, during load carriage, the lower extremity muscles including the plantar flexors must generate more force to offset the added demand to support the extra weight^29^. Adding stiffness to the foot during these activities could potentially improve locomotion economy, and we will conduct future studies to test this hypothesis. These efforts will continue to address the fundamental structure -function relationships of the human foot and ankle, and may provide new insights for augmenting human performance or restoring normal locomotion in persons with impaired foot and ankle functions.

This study has a few notable limitations. First, our approach to increase midsole longitudinal bending stiffness affected both the foot’s energy dissipation and the lever arm of the ground reaction force about the ankle. Thus, the present study cannot separate their independent effects, and a future study that involves footwear of varying stiffness and geometry may be needed. Another potential confounding variable that makes it difficult to isolate the effects of added foot stiffness on metabolic cost is the added mass of the shoes and insoles^28^,^43^. In this study, we did not control for the added mass by the shoe and/or the insoles since we originally expected that the metabolic cost could be reduced *despite* added mass. We therefore computed an ‘adjusted’ whole-body net metabolic power to account for the mass differences across all added foot stiffness conditions based on published results from Browning *et al*.^28^, and we found that the greatest stiffness condition still had greater metabolic cost relative to all other conditions. Thus, it is likely that added mass at the foot did not alter the overall conclusion of this study.

Our method for quantifying soleus fascicle force also has a few limitations. The analysis is based on assumptions that the force is proportional to the physiological cross-sectional area, and that relative muscle activations among all plantar flexor muscles are similar. The change in muscle activation across different added foot stiffness conditions was similar for three of the four recorded muscles (soleus, lateral gastrocnemius, and tibialis anterior), as added foot stiffness significantly increased activation of these muscles (Supplementary Fig. S3). Additionally, our estimates of soleus force assume that the contributions of the antagonistic muscles such as tibialis anterior are negligible, and significant activity of these muscles would cause underestimates of actual soleus force. Even though the tibialis anterior activity was greater at the greater added foot stiffness conditions, the increased activation occurred primarily during early stance phase and not during push-off (Supplementary Fig. S3). Furthermore, increase in tibialis anterior activation would only underestimate the soleus force at greater added foot stiffness conditions, which would actually serve to strengthen the overall findings that added foot stiffness increased soleus force. For this study, we were mainly interested in the change in force across different foot stiffness conditions, rather than the absolute magnitude of force. We are confident that the inherent limitations of the soleus fascicle force estimates do not affect the overall findings of this study.

## Conclusions

In this study, we manipulated the dual function of the foot (a lever for gearing and a damper for energy dissipation) by adding stiffness with shoes and insoles. These altered foot/shoe/insole structural properties modulated the force generating capacity of the soleus muscle. With added foot stiffness, soleus force output and force per unit activation were enhanced, likely aided by reduced fascicle shortening velocity. Despite this shifting of force-velocity behaviour of the soleus, whole-body metabolic energy expenditure increased when walking with an extremely stiff foot/shoe combinations. This elevated metabolic cost is likely due to the added force demand of the plantar flexors, as walking on a rigid foot-to-ground interface compromises plantar flexor mechanical advantage.

## Methods

### Adding Stiffness to the Foot with Shoes and Insoles

To increase bending stiffness of the foot/shoe complex, we designed carbon fibre insoles with three different thicknesses: 0.8, 1.6, and 3.2 mm. These custom insoles were designed to fit within a standardized shoe (New Balance 1400), with sizes ranging from female 37 (Euro) to male 46, or length of 23.5 to 29.4 cm. The custom insoles replicated the shape of the shoe’s original insoles such that the custom insoles could be placed underneath the shoe insoles while walking. To quantify the shoe and insole longitudinal bending stiffness, we used an instrumented mechanical testing apparatus (Mechanical Testing Systems, MN, USA) to perform a three-point bending analysis described in previous studies^20^,^21^. Specifically, the shoe was placed in a frame with two supporting bars 8.0 cm apart under its forefoot. A vertical displacement was applied cyclically (20 cycles at a rate of 15 mm/s) to the dorsal surface of the shoe midway between the two supporting bars, inducing bending in the metatarsal-phalangeal region of the shoe. Bending stiffness of the shoes and shoes with insoles (N/mm) was estimated by the slope of the linear regression of the force-displacement data (collected at 100 Hz) over the displacement range from 5 to 10 mm during loading. We chose this range of loading, as opposed to the 5 to 6 mm of displacement used in previous studies^20^,^21^, because the deflection of the carbon fibre insoles was more pronounced during this displacement range. A linear regression for bending stiffness estimation was justified because the coefficient of determination (R^2^) of the line of best-fit was greater than 0.98 for all of the shoes and insoles tested.

### Experimental Protocol

**Figure 6:**
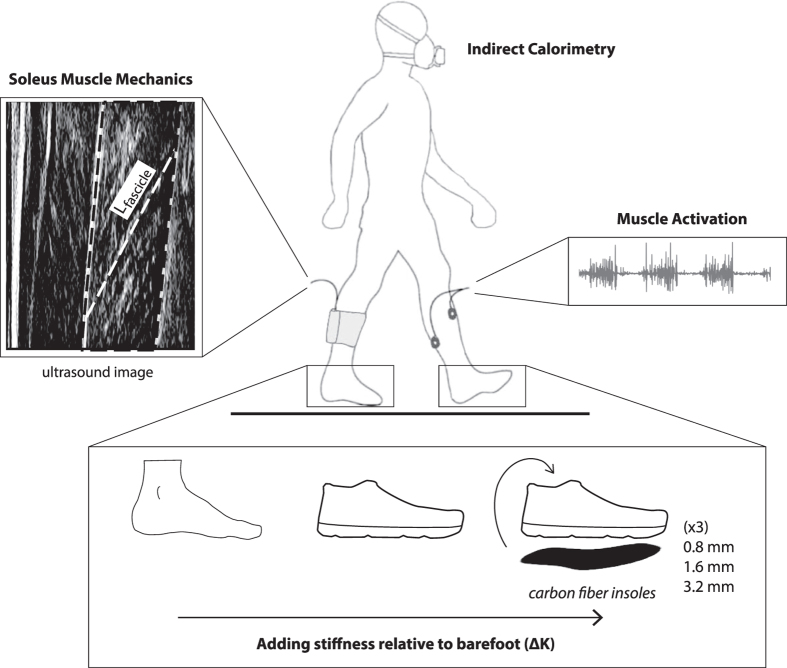
Experimental protocol. Each subject walked on an instrumented treadmill at 1.25 m/s under 5 conditions in a randomized order: barefoot, shod, and shod with three levels of added insole thickness. Data collection was separated in two testing sessions separated by approximately 24 hours: one day for metabolic energy analysis, and another day for lower limb mechanics including ultrasound and electromyography. The order of the days was randomized. Rate of whole-body metabolic energy expenditure was analysed using indirect calorimetry. B-mode ultrasound images were acquired from the right soleus muscle, while electromyography sensors were placed on the ankle muscles of the left limb including lateral and medial gastrocnemius, soleus, and tibialis anterior.

The protocol was approved by the Institutional Review Board (IRB) of the University of North Carolina at Chapel Hill, USA. Twenty healthy subjects (10 females and 10 males; ages 24.2 ± 1.1 years, mass 74.0 ± 3.5 kg and height 1.73 ± 0.02 m; mean ± standard error) signed an informed consent to participate in this study. The methods were carried out in accordance with the IRB-approved protocol. The subjects walked on an instrumented treadmill (Bertec, Columbus, OH, USA) on two separate days: one day to collect metabolic energy expenditure, and the other to analyse lower limb neuromechanics that included muscle activation, foot mechanics, and soleus muscle fascicle behaviour (Fig. 6). The order of the days was randomized, with the two testing days separated by approximately 24 hours. On each testing day, subjects walked at 1.25 m/s under five different foot conditions in a randomized order: barefoot (ΔK = 0), shod (ΔK = 14.8 ± 0.5 N/mm), shod with 0.8 mm insole (ΔK = 22.5 ± 0.5 N/mm), shod with 1.6 mm insole (ΔK = 28.7 ± 0.8 N/mm), and shod with 3.2 mm insole (ΔK = 65.6 ± 2.9 N/mm).

### Analysis: Metabolic Energy Expenditure using Indirect Calorimetry

A portable metabolic measurement system (Oxycon Mobile, Viasys Healthcare, CA, USA) was used to acquire rates of oxygen consumption and carbon dioxide production. All walking trials lasted seven minutes, and averaged data from the last two minutes were used to estimate rates of metabolic energy expenditure using a standard equation^44^. Net metabolic power (normalized by body mass) during the walking trials was estimated by subtracting the metabolic power during 7 minutes of quiet standing^29^.

### Analysis: Lower Limb Mechanics

An eight-camera motion analysis system (Vicon, Oxford, UK) was used to capture lower-limb kinematic data (250 Hz) and an instrumented treadmill was used to collect kinetic data (1000 Hz). A six degree-of-freedom market set^45^ was used to track lower extremity motion. A 2^nd^ order dual-pass low-pass Butterworth filter was applied to the kinematic (6 Hz) and kinetic (25 Hz) data.

### Analysis: Foot Deformation Power

Foot deformation power (P_ftd_) was quantified using a deforming segment model described previously^14^,^15^,^19^. Briefly, this technique encompasses the movement of the forces underneath the foot to quantify the rate of energy stored, released, generated, and/or dissipated. Specifically, P_ftd_ is calculated as:


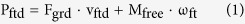


where F_grd_ is the ground reaction force, v_ftd_ is the velocity of foot deformation (or the velocity of the centre-of-pressure in the foot’s reference frame), M_free_ is the free moment of the ground reaction force, and ω_ft_ is the angular velocity of the foot segment. v_ftd_ is calculated as:


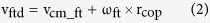


where v_cm_ft_ is the centre-of-mass of the foot, and r_COP_ is the vector from the foot’s centre-of-mass to the centre-of-pressure. To visualize how the forces propagate underneath the foot, the centre-of-pressure data were transformed in a foot-based coordinate system^3^ (Fig. 1).

We use the terms ‘foot deformation’ loosely, as foot deformation power could be attributed to muscle-tendon structures of the foot^46^,^47^, soft tissue deformation^19^, or non-anatomical structures^15^,^48^ that may include shoe and insole contributions. The negative and positive work performed by these foot structures was determined by taking the time-integral of the negative and positive portions of foot deformation power, respectively. Net work was calculated as the sum of negative and positive work.

### Analysis: Gear Ratio

Gear ratio during stance was calculated as the ratio of the lever arm of the ground reaction force (relative to the ankle joint centre, in the sagittal plane) and the moment arm of the plantar flexor muscle-tendon unit. To estimate changes in plantar flexor moment arm during walking, we developed a model-based estimate of moment arm as a function of ankle dorsi-plantar flexion angle^49^. This model was derived by combining: (1) subject-specific anthropometric measurements obtained when the ankle was at a neutral angle, and (2) generic regression model of moment arm as a function of dorsi-plantar flexion angles, based on published *in-vivo* data derived from magnetic resonance imaging^50^. Subject-specific anthropometry was obtained by capturing a digital photograph of the subjects’ right foot as the subject stood at a neutral 90 degree ankle angle on a reference block (Supplementary Fig. S4). This block served as a calibration object to convert pixel coordinates of the photograph to metric units. Using a custom-written digitizing software (Mathworks, MA, USA), we digitized two anatomical landmarks: the lateral malleolus and the posterior aspect of the Achilles tendon at the same height as the malleolus. The distance between these two landmarks defined the plantar flexor moment arm at a neutral ankle angle. This derived subject-specific moment arm was divided by the moment arm estimated at neutral ankle angle using the generic regression model with data from Maganaris *et al*.^50^, to give a moment arm scaling factor. This scaling factor was then used to convert the generic model estimates to a subject-specific prediction of plantar flexor moment arm as a function of ankle dorsi-plantar flexion angles. Using this model, the plantar flexor moment arm during stance phase of walking was estimated based on the ankle angle measurement from the motion capture system. Generally, the plantar flexor moment arm decreases as ankle dorsi flexion angle increases^50^.

The peak gear ratio and the average gear ratio were calculated from 5–95% of stance^22^. This interval was chosen because the estimates of ground reaction force lever arm are susceptible to error at low magnitude of ground reaction force^22^.

### Analysis: Electromyography

Surface electromyography (EMG) data were recorded (Biometrics, Newport, UK) from the soleus (SOL), medial gastrocnemius (MG), lateral gastrocnemius (LG), and tibialis anterior (TA) of the left leg only. Only the soleus EMG data are reported in the main text, and the remaining EMG data are included as Supplementary data (Supplementary Fig. S3). The EMG signals were high-pass filtered with 2^nd^ order dual-pass Butterworth filter (20 Hz), rectified, and low-pass filtered with a 2^nd^ order dual-pass Butterworth filter (10 Hz) to create a linear enveloped EMG. The time integral of the processed EMG data was computed during the stance phase of walking, when the vertical ground reaction force exceeded 20 N. EMG data from one subject were omitted due to technical difficulties.

### Analysis: Soleus muscle fascicle behaviour using ultrasonography

A flat-shaped linear ultrasound probe (LV 7.5/60/96Z; Telemed, Lithuania) was used to acquire images of soleus muscle fascicle from the right leg (sampled at 80 Hz). The probe was placed over the medial gastrocnemius, and the probe placement was adjusted until the soleus fascicles were visible from superficial to deep aponeurosis. The probe was secured using a self-adhesive elastic wrap. Accuracy and reliability of this technique for fascicle length measurements have been described in previous studies^51^,^52^,^53^. To quantify fascicle length and pennation angle changes, a previously validated automatic tracking software^54^,^55^ along with an algorithm to account for temporal drift^56^ were used. If the tracking algorithm was unable to define the ends of the fascicles, we manually corrected the fascicle endpoints. All images were visually inspected to verify accurate tracking. Soleus fascicle velocity was calculated by differentiating the fascicle length with respect to time.

To quantify soleus fascicle force, we used an approach similar to Farris *et al*.^31^. An estimate of net ankle moment (derived from inverse dynamics) was divided by the subject-specific plantar flexor moment arm to compute plantar flexor force. The soleus contribution to this force was estimated using physiological cross-sectional area of the soleus relative to all other plantar flexor muscles (scaling factor of 0.54)^57^. Finally, the soleus muscle-tendon force was then divided by the cosine of the soleus pennation angle to estimate the soleus fascicle force.

To relate soleus fascicle force and muscle activation, we computed force per unit activation, which is the ratio of integrated force to integrated EMG during stance. Soleus kinetic measures were obtained from the right leg and EMG measures were acquired from the left leg, because the ultrasound probe prevented adequate placement of EMG electrodes on the same leg. Thus, we assumed symmetry between limbs.

### Statistical Tests

The main outcome variables for this study included: mechanical work due to foot deformation (negative, positive, and net work), peak and average gear ratio, soleus fascicle force (integrated force during stance and peak during stance), fascicle length (average length during stance and instantaneous length at the time of peak force), fascicle velocity (average velocity during stance and instantaneous velocity at the time of peak force), soleus muscle activation (integrated EMG during stance, and peak), force per unit activation, and whole-body net metabolic power. We used a one-factor repeated-measures ANOVA to assess the effects of added foot stiffness on the main outcome variables. The level of statistical significance was set at α = 0.05. When a significant main effect was found, Tukey-corrected *post hoc* tests were used to make pairwise comparisons across the foot conditions. All values presented in this study are the mean plus and minus the standard error of the mean, unless otherwise noted.

In all figures (Figs 1, 2, 3, 4, 5), the reported p-values signify the main effect of added foot stiffness on the outcome variables. Additionally, we reported significant pair-wise comparisons on each figure with annotations. When one foot stiffness condition was significantly different with respect to each of the other conditions, we used the symbol (**). All additional significant pair-wise comparisons were annotated with a square bracket symbol.

## Additional Information

**How to cite this article**: Takahashi, K. Z. *et al*. Adding Stiffness to the Foot Modulates Soleus Force-Velocity Behaviour during Human Walking. *Sci. Rep.* **6**, 29870; doi: 10.1038/srep29870 (2016).

## Supplementary Material

Supplementary Information
