# Supplementary material for: Adding Stiffness to the Foot Modulates Soleus Force-Velocity Behaviour during Human Walking
Source: Sci Rep. 2016 Jul 15;6:29870. doi: 10.1038/srep29870 (PMC4945910; doi:10.1038/srep29870)
Supplement: Supplementary Information [file srep29870-s1.pdf]

Supplementary Information

Adding Stiffness to the Foot Modulates Soleus Force-Velocity Behaviour during Human Walking

Kota Z. Takahashi

Michael T. Gross

Herman van Werkhoven

Stephen J. Piazza

Gregory S. Sawicki

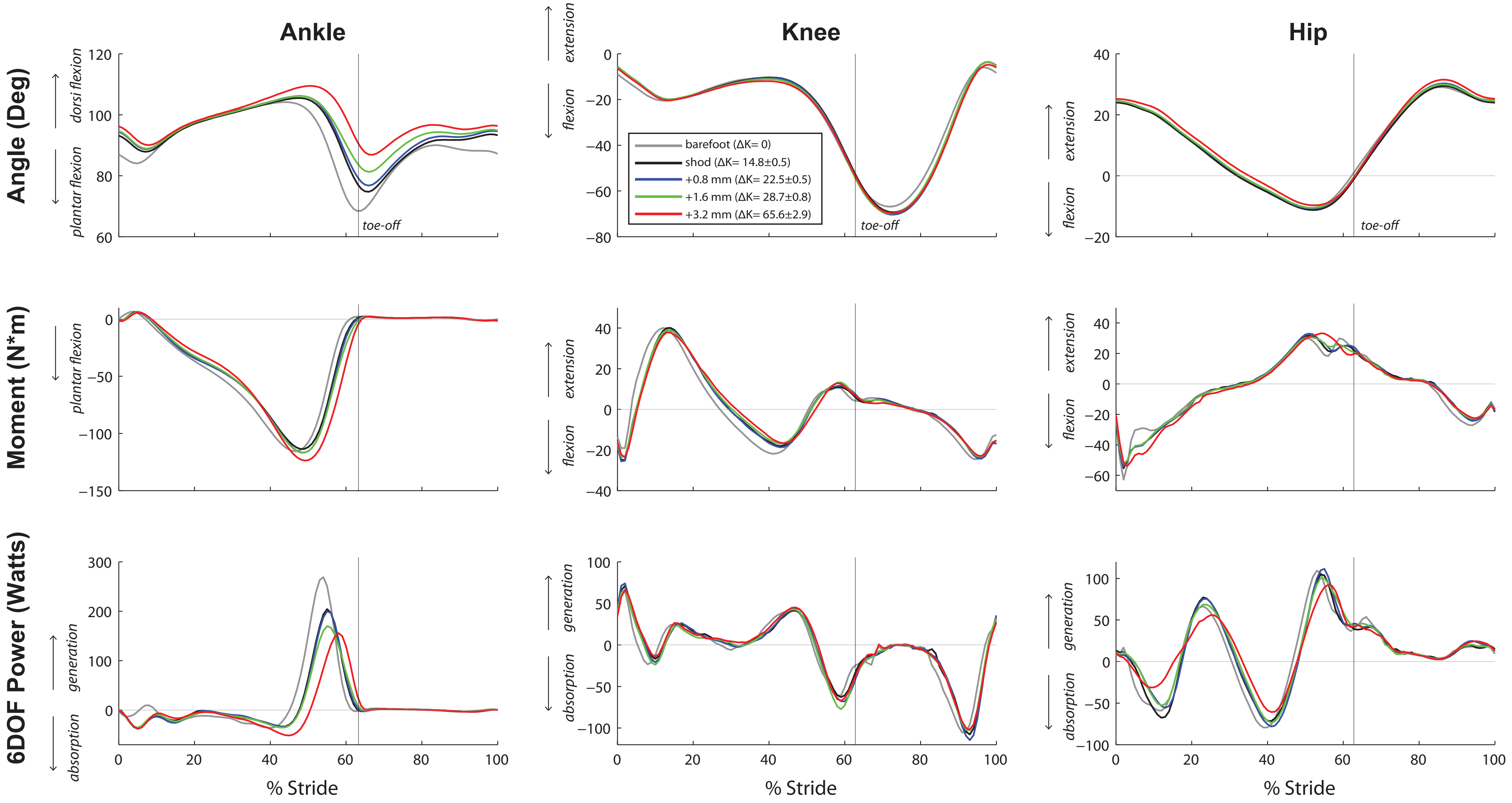

Supplementary Figure S1: Joint angle, moment and power profiles of the ankle, knee and hip.
Time-normalized data (stride cycle) of joint angle, moment and power data of the ankle, knee and hip (N = 20, right limb).
Joint power was estimated using a 6 degree-of-freedom (DOF) analysis (Buczek et al., 1994; Zelik et al., 2015).

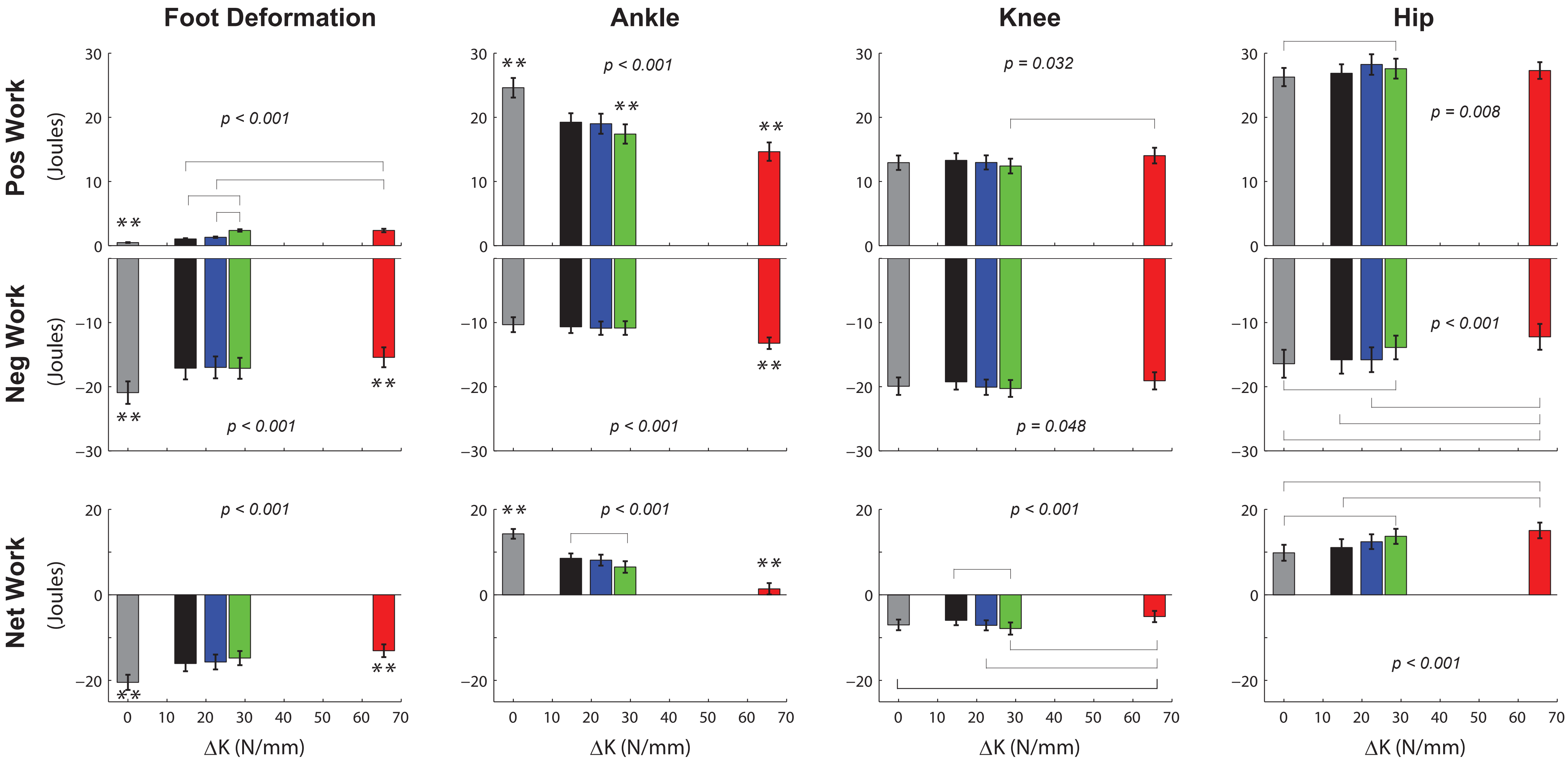

Supplementary Figure S2: Work profiles of the foot, ankle, knee and hip.
Total positive work, negative work, and net work were quantified during stride (N = 20, mean ± s.e.m).
Adding stiffness to the foot influenced the mechanical work done by the muscle-tendon structures crossing the ankle, knee, and hip, as well as through foot deformation. In particular, increased stiffness decreased the magnitude of net work due to foot deformation ($p < 0.001$) and decreased the magnitude of positive work done by the muscle-tendon structures of the ankle joint ($p < 0.001$).

The p-values indicate a significant main effect of added foot stiffness.
**denotes significant pair-wise comparisons with respect to each of the other conditions, and square brackets indicate additional significant pair-wise comparisons.

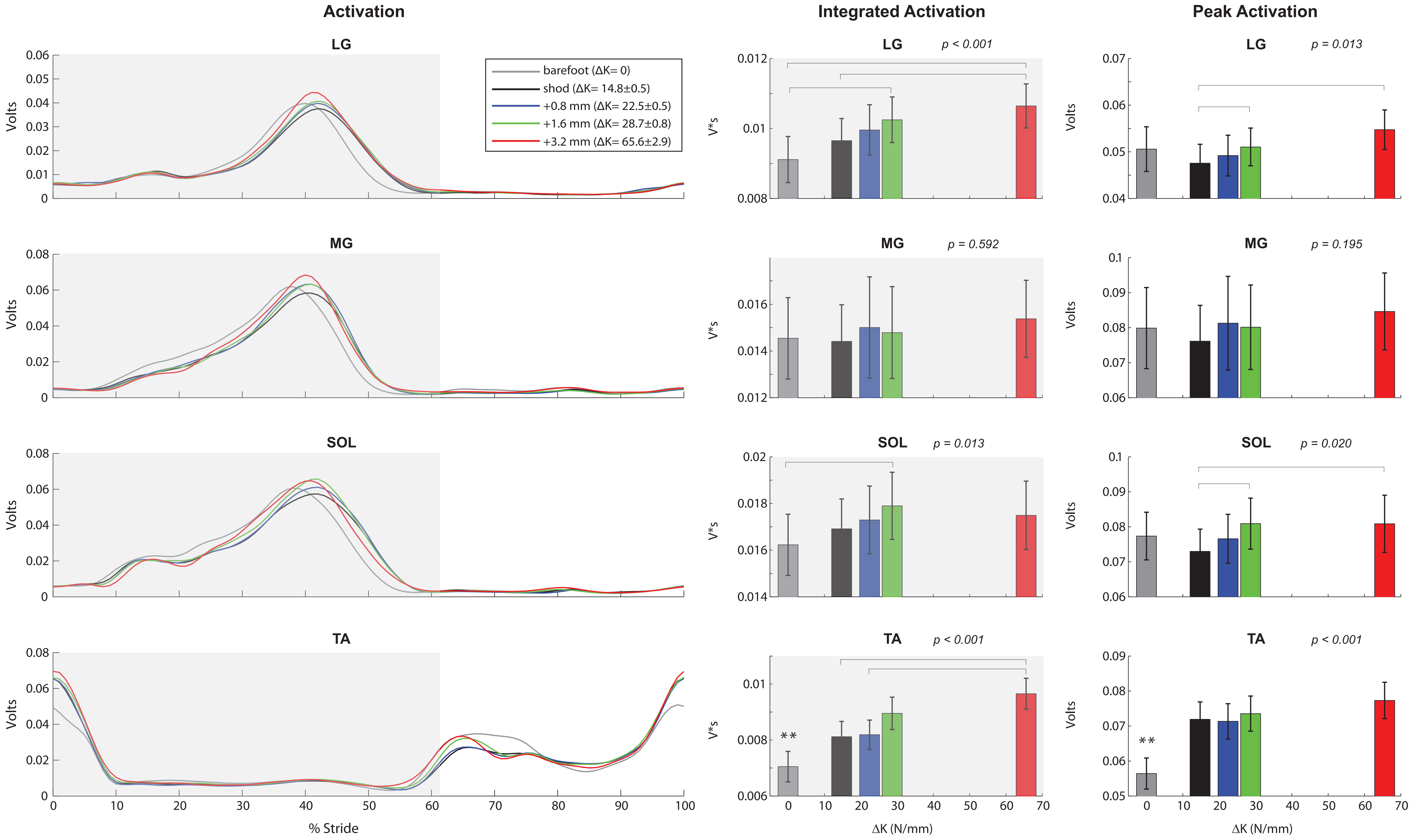

Supplementary Figure S3: Electromyography data of the ankle muscles.

Time-normalized data (stride cycle) of activation of lateral gastrocnemius (LG), medial gastrocnemius (MG), soleus (SOL) and tibialis anterior (TA) (N = 19, left limb). Stance phase is highlighted in grey.
Adding stiffness to the foot increased integrated activation during stance for LG ($p < 0.001$), SOL ($p = 0.013$), and TA ($p < 0.001$).
Adding stiffness also increased peak activation for LG ($p = 0.013$), SOL ($p = 0.020$) and TA ($p < 0.001$).

The p-values indicate a significant main effect of added foot stiffness.
**denotes significant pair-wise comparisons with respect to each of the other conditions, and square brackets indicate additional significant pair-wise comparisons.

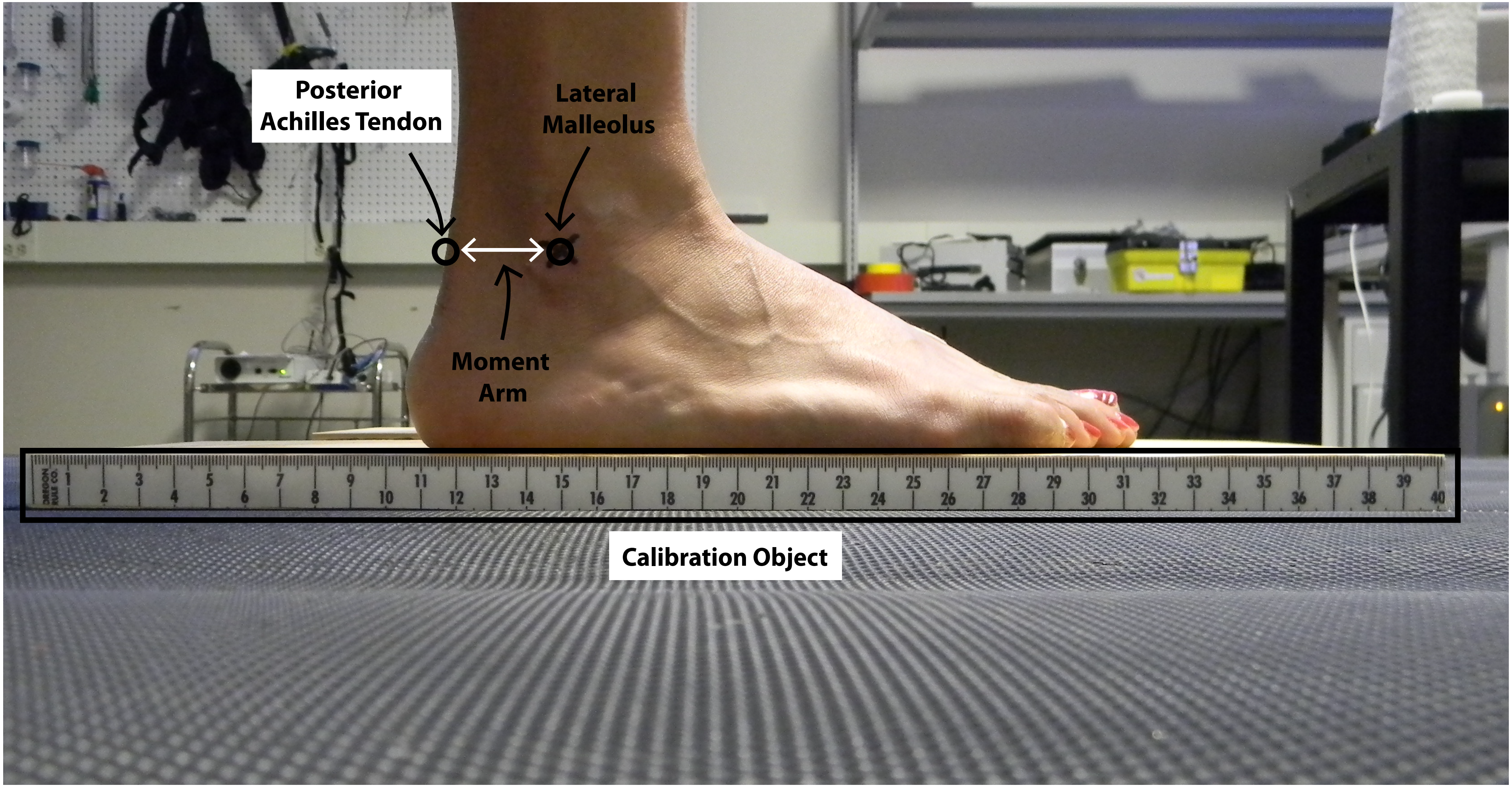

Supplementary Figure S4: A photography-based method to estimate plantar flexor moment arm at a neutral ankle angle.

Subject-specific plantar flexor moment arm was estimated by capturing a digital photograph of the subject's right foot as the subject stood at a neutral 90 degree ankle angle on top of a reference block. This block served as a calibration object to convert pixel coordinates of the photograph to metric units. Two landmarks were identified using a custom-written digitizing software: the lateral malleolus and the posterior aspect of the Achilles tendon at the same height as the malleolus. The distance between these two landmarks defined the plantar flexor moment arm at the neutral ankle angle.
